# Cystic Fibrosis Foundation Evidence-Based Guideline for the Management of CRMS/CFSPID

**DOI:** 10.1542/peds.2023-064657

**Published:** 2024-05-01

**Authors:** Deanna M. Green, Thomas Lahiri, Karen S. Raraigh, Fadel Ruiz, Jacquelyn Spano, Nicholas Antos, Lynn Bonitz, Lillian Christon, Myrtha Gregoire-Bottex, Jaime E. Hale, Elinor Langfelder-Schwind, Álvaro La Parra Perez, Karen Maguiness, John Massie, Erin McElroy-Barker, Meghan E. McGarry, Angelique Mercier, Anne Munck, Kathryn E. Oliver, Staci Self, Kathryn Singh, Michael Smiley, Steven Snodgrass, Audrey Tluczek, Pamela Tuley, Paula Lomas, Elise Wong, Sarah E. Hempstead, Albert Faro, Clement L. Ren

**Affiliations:** a Johns Hopkins All Children’s Hospital, St Petersburg, Florida; b University of Vermont Children’s Hospital, Burlington, Vermont; c Johns Hopkins University School of Medicine, Baltimore, Maryland; d Baylor College of Medicine, Houston, Texas; e Stanford University School of Medicine, Stanford, California; f Medical College of Wisconsin, Children’s Wisconsin, Milwaukee, Wisconsin; g Cohen Children’s Medical Center of NY/Northwell Health, New Hyde Park, New York; h Medical University of South Carolina, Charleston, South Carolina; i Advanced Pediatric Pulmonology, Pllc, Miramar, Florida; j Memorial Health Network, Hollywood, Florida; k University of Massachusetts Chan Medical School, Worcester, Massachusetts; l Lenox Hill Hospital/Northwell Health, New York, New York; m John B. Goddard School of Business and Economics, Weber State University, Ogden, Utah; n Riley Hospital for Children at IU Health, Indianapolis, Indiana; o University of Melbourne Murdoch Childrens Research Institute, Melbourne, Australia; p Rutgers Robert Wood Johnson Medical School, New Brunswick, New Jersey; q University of California San Francisco, San Francisco, California; r Robert H. Lurie Children’s Hospital of Chicago, Northwestern University, Feinberg School of Medicine, Chicago, Illinois; s Hospital Necker Enfants malades, AP-HP, Paris, France; t Emory University School of Medicine, Atlanta, Georgia; u University of Alabama at Birmingham, Birmingham, Alabama; v University of California, Irvine, Orange, California Miller Children’s and Women’s Hospital, Long Beach, California; w Saint Louis University, St Louis, Missouri; x Prisma Health Children’s Hospital, Greenville, South Carolina; y University of Wisconsin-Madison, Madison, Wisconsin; z Texas Children’s Hospital, Houston, Texas; aa The Cystic Fibrosis Foundation, Bethesda, Maryland; bb The Children’s Hospital of Philadelphia, Philadelphia, Pennsylvania

## Abstract

A multidisciplinary committee developed evidence-based guidelines for the management of cystic fibrosis transmembrane conductance regulator-related metabolic syndrome/cystic fibrosis screen-positive, inconclusive diagnosis (CRMS/CFSPID). A total of 24 patient, intervention, comparison, and outcome questions were generated based on surveys sent to people with CRMS/CFSPID and clinicians caring for these individuals, previous recommendations, and expert committee input. Four a priori working groups (genetic testing, monitoring, treatment, and psychosocial/communication issues) were used to provide structure to the committee. A systematic review of the evidence was conducted, and found numerous case series and cohort studies, but no randomized clinical trials. A total of 30 recommendations were graded using the US Preventive Services Task Force methodology. Recommendations that received ≥80% consensus among the entire committee were approved. The resulting recommendations were of moderate to low certainty for the majority of the statements because of the low quality of the evidence. Highlights of the recommendations include thorough evaluation with genetic sequencing, deletion/duplication analysis if <2 disease-causing variants were noted in newborn screening; repeat sweat testing until at least age 8 but limiting further laboratory testing, including microbiology, radiology, and pulmonary function testing; minimal use of medications, which when suggested, should lead to shared decision-making with families; and providing communication with emphasis on social determinants of health and shared decision-making to minimize barriers which may affect processing and understanding of this complex designation. Future research will be needed regarding medication use, antibiotic therapy, and the use of chest imaging for monitoring the development of lung disease.

Cystic fibrosis (CF) newborn screening (NBS) has been offered in many countries around the world and in every US state since 2010.^1^ An unintended consequence of CF NBS is the detection of infants with an abnormal CF NBS result but inconclusive diagnostic testing, which has been termed CF transmembrane conductance regulator (*CFTR*)-related metabolic syndrome (CRMS) in the United States and CF screen-positive, inconclusive diagnosis (CFSPID) in other parts of the world.^2^ Many people with CRMS/CFSPID are healthy, but a small proportion (<10%) will be reclassified as CF because of an updated annotation of their *CFTR* variants as CF-causing or an increase in sweat chloride concentration (sweat [Cl^−^]) to ≥60 mmol/L(2). Approximately 10% will also develop clinical features that are concerning for CF (eg, pulmonary disease, *Pseudomonas aeruginosa* [*Pa*] in a respiratory culture).^2^

In 2009, the Cystic Fibrosis Foundation (CFF) convened an expert panel to develop consensus recommendations for the management of infants with CRMS.^3^ There have been multiple studies of CRMS/CFSPID outcomes and genetics^2,4^ since then; thus, a diverse committee of CF providers and parents of people with CRMS/CFSPID was assembled in 2021 to develop an up-to-date, evidence-based guideline for the management and care of people with CRMS/CFSPID.

This guideline is intended to be used by both CF specialists and primary care providers (PCPs) who care for people with CRMS/CFSPID and their families. It should supplement the standard care provided in primary care. The guideline will not address how CRMS/CFSPID is diagnosed, nor the criteria for reclassifying people with CRMS/CFSPID as people with CF (pwCF) because these recommendations already exist;^2,5^ however, it will address genetic testing to better refine the diagnosis. The European Cystic Fibrosis Society (ECFS) recently published a consensus guidance document concerning the management of children with CRMS/CFSPID.^6^ The current guideline is evidence-based and is intended to complement the ECFS paper.

CFF intends for this guideline to summarize data and provide reasonable clinical recommendations to clinicians, patients, and other stakeholders. The application of these recommendations should not be mandated. Care decisions regarding individual patients should be made by using a combination of these recommendations, an associated benefit–risk assessment of the treatment options, the patient’s individual and unique circumstances, and the goals and preferences of the patients and families that the team serves as a part of shared decision-making (SDM) between the patient and clinician.

## METHODS

CFF sponsored the creation of the committee. The committee defined people with CRMS/CFSPID as people with an abnormal CF NBS result and (1) a sweat [Cl^−^] of <30 mmol/l (normal) and 2 *CFTR* variants, at least 1 of which with unclear phenotypic consequences, or (2) sweat [Cl^−^] of ≥30 to 59 mmol/L (intermediate value) and 1 or no CF-causing variants.^2^ An online survey was sent to CF care centers (CFCC) and the families of people with CRMS/CFSPID to identify high-priority issues for both groups. Based on survey results, input from the committee, and areas of further research identified in previous guidelines, 24 questions were written in patient, intervention, comparison, outcome (PICO) format.^7^ A systematic review was performed by using PubMed and the Cumulative Index to Nursing and Allied Health Literature databases. Literature review and evidence grading were performed by 4 working groups: genetic testing, monitoring, treatment, and psychosocial and communication issues. The groups generated recommendations that were graded by using the US Preventive Services Task Force (USPSTF) definitions (Table 1).^8^ The committee adhered to the USPSTF Procedure Manual^9^ in generating and reviewing 31 specific recommendations. Statements that received ≥80% consensus among the committee were approved, resulting in 30 final recommendations (Table 2) and 1 non-consensus statement (Table 3). Details of the committee selection, PICO framework, search terms, and recommendation statements are available in the Supplemental Information and Supplementary Table 5.

## RECOMMENDATIONS

### Genetic Testing

#### Additional *CFTR* Genetic Testing

1. The CFF recommends that people with CRMS/CFSPID who have <2 disease-causing variants identified by NBS should undergo sequencing of the coding and flanking regions and deletion/duplication (del/dup) analysis of the coding and exon flanking regions of *CFTR* (Grade B).

Establishing the *CFTR* genotype of people with CRMS/CFSPID is important for diagnosis, monitoring, and genetic counseling and, should reclassification to CF occur, may also be useful for the approval of *CFTR* modulators. Genotyping requires a step-wise approach that is dependent on the state’s NBS algorithm. A glossary of genetic terms is available in Supplemental Table 6. Further genetic analysis is not necessary when 2 *CFTR* variants identified by NBS are confirmed to be *in trans* (1 inherited from each parent). However, sequencing of the coding and flanking intronic regions of *CFTR*, with del/dup analysis to evaluate for large structural variants, such as exon deletions, is useful in the diagnostic workup.^10^ It should be performed when people with CRMS/CFSPID have only 1 or no identified disease-causing *CFTR* variants from NBS.

Variant panels commonly used in NBS are typically limited to well-established CF-causing variants and may not identify variants of varying clinical consequence (VVCCs).^1,11^ The identification of VVCCs may inform the future risk of the development of symptoms related to *CFTR* dysfunction and refine the reproductive risks of parents and other family members.^12^ Studies of people with CRMS/CFSPID who have 1 variant identified by NBS panel (often F508del [c.1521_1523del; p.Phe508del]) have revealed that sequencing identified a second variant in 31% to 90% of cases^13–17^ supporting our recommendation to perform further *CFTR* variant testing in these infants. Regularly monitoring databases such as CFTR2 (www.cftr2.org) and ClinVar (https://www.ncbi.nlm.nih.gov/clinvar/) can be helpful to stay updated on the annotation status of *CFTR* variants.

- The CFF recommends, for people with CRMS/CFSPID, selectively offering full-gene *CFTR* sequencing including intronic regions when the *CFTR* genotype remains incomplete after coding and flanking region sequencing and del/dup (Grade C).

*CFTR* sequencing that includes all intronic regions has identified putatively causal variants among individuals with CF, *CFTR*-related disorders, and positive CF NBS results.^18–23^ Tests that include many flanking nucleotides and several deep intronic variants are fairly comprehensive; additional testing of all other intronic regions may offer little additional value or sensitivity. Full intronic sequencing should be selectively performed in people with CRMS/CFSPID who demonstrate evidence of potential *CFTR* dysfunction (eg, sweat [Cl−] of 30–59 mmol/L), or individuals for whom the clinical suspicion of CF development is high and who possess only 1 previously identified causal variant after full sequence and del/dup is provided (see Supplemental Information for further discussion). Availability of full *CFTR* sequencing is currently limited; this service may become more widely available in the future.

#### CFTR Testing in Family Members

- The CFF recommends *CFTR* genetic evaluation for parents of people with CRMS/CFSPID when phasing the *CFTR* variants (ie, in *cis* or *trans*) would inform the diagnostic status of the individual by confirming the inheritance pattern (Grade A).
- The CFF recommends offering *CFTR* genetic evaluation for siblings of people with CRMS/CFSPID (Grade B).

For people with CRMS/CFSPID who have 2 identified *CFTR* variants, determining if the variants reside within the same copy (in *cis*) or different copies (in *trans*) of *CFTR* is achieved through the genetic testing of at least 1 first-degree relative, which is referred to as “phasing.” *CFTR* variants in *cis* can lead to diagnosis of CF carrier instead of CRMS/CFSPID,^21,24^ which bears health and reproductive implications for the individual and family members.

Evaluating the siblings of people with CRMS/CFSPID can identify at-risk individuals who may also benefit from clinical monitoring and follow-up.^25,26^ This assessment should be offered to families and promotes SDM with emphasis on the risks and benefits to the individual sibling.^6^ In many instances, this may be delayed until childbearing age and at a time the sibling may also share in the decision-making regarding the value of this genetic information.

#### Genetic Counseling

- The CFF recommends, for families of people with CRMS/CFSPID, that health care professionals (HCPs) providing genetic counseling should have training or clinical expertise in CF and genetics. A licensed or certified genetic counselor (GC) should be accessible to families of people with CRMS/CFSPID for further support, including discussions regarding future reproductive decision-making (Grade B).

Published recommendations affirm that genetic counseling should be offered to the families of people with CRMS/CFSPID.^6,27,28^ The genetic counseling provider, whether they are a licensed or certified GC or a CF clinician, should have a high level of expertise in both CF genetics and CRMS/CFSPID.^27^ Some families feel more comfortable discussing these topics with providers outside the CFCC.^29,30^ Having access to a trained GC to discuss genetic findings and complement team members in providing psychosocial support promotes understanding^3,6^ and strengthens long-term retention of genetic knowledge.^31^ Genetic counseling at regular intervals throughout the lifespan allows for timely, accurate, supportive, and non-directive information on recurrence risk and reproductive options.^29^

### Monitoring

#### Frequency of Follow-Up

- The CFF recommends, for people with CRMS/CFSPID, at least annual follow-up by a CF clinician and nurse, with an initial assessment to include a social worker, a mental health coordinator (MHC), and/or a genetic counseling provider. Continued follow-up by a social worker, MHC, and/or genetic counselor should be part of the care of CRMS/CFSPID, depending on the needs of that individual and family (Grade B).

The primary goal of monitoring people with CRMS/CFSPID is to detect those individuals who may be reclassified as CF and would benefit from care at a CFCC. Equally important is avoiding the overmedicalization of otherwise healthy individuals, which involves its own set of adverse consequences. Families should be informed about possible outcomes for people with CRMS/CFSPID and plans for future monitoring visits. Communication with PCPs is essential and should highlight reasons for more frequent reassessment by CFCC (eg, persistent cough, constipation, or inadequate weight gain). Routine follow-up should be provided by clinicians with expertise in CF and *CFTR* genetics to reexamine *CFTR* variants (eg, periodic assessment of the CFTR2 database) and changes in clinical status. Because most people with CRMS/CFSPID are healthy, evaluation by an entire multidisciplinary team is often unnecessary.

#### Sweat Chloride Testing (SCT)

- The CFF recommends for people with CRMS/CFSPID to repeat SCT at 6 months of life and annually, at least until age 8 years (Grade B).

SCT is the mainstay for diagnosing CF and part of the diagnostic criteria for CRMS/CFSPID. One reason for reclassification of CRMS/CFSPID to CF is a sweat [Cl^−^] of >60 mmol/L. The authors of multiple studies have reported sweat [Cl^−^] elevation above this level after an initial sweat [Cl^−^] of <60 mmol/L during the newborn period.^16,30,32–39^ However, evidence is lacking regarding changes in sweat [Cl^−^] after 8 years of age, and SDM with parents should be used to determine if SCT should continue past this age. Careful consideration for continued SCT may be given to certain populations, including (1) individuals with initial sweat [Cl^−^] of 40 to 59 mmol/L because they are up to 10 times more likely to have a sweat [Cl−] elevation of >59 mmol/L in later childhood^33,40^ and (2) individuals with sweat [Cl^−^] increasing at a high rate over time (>5 mmol/L per year).^16^

#### Respiratory Cultures

- The CFF recommends, for people with CRMS/CFSPID, selectively offering CF respiratory cultures at each visit (at least until age 8 years) and as clinically indicated for respiratory symptoms (Grade C).

Airway infection with CF-associated microorganisms, specifically *Pa*, is considered a phenotypic feature supporting a CF diagnosis. Compared with the general population, people with CRMS/CFSPID exhibit a higher prevalence of *Pa* (10.7% to 78.6%), *Stenotrophomonas maltophilia* (4.9% to 10%), and *Staphylococcus aureus* (40% to 85%) in respiratory cultures.^30,41,42^ Some studies have revealed that people with CRMS/CFSPID who reclassify to CF more likely have a positive culture result for *Pa* than those who do not reclassify (33% vs 10%), whereas other reports have revealed no difference.^30,35^ However, a positive culture result alone does not warrant reclassification. Some families and physicians may not want to expose the individual to trauma associated with cultures. Respiratory culture data from people with CRMS/CFSPID >8 years of age are currently lacking. If persistent respiratory symptoms are present, obtaining respiratory cultures in people with CRMS/CFSPID may be warranted. Thus, the evidence supports that cultures obtained via throat swab or sputum should be selectively offered after SDM with the family.

#### Laboratory Testing

- The CFF recommends, for people with CRMS/CFSPID, that measurement of fecal elastase (FE) should be performed at the initial assessment. Further testing of FE can be provided when clinically appropriate (Grade B).
- The CFF recommends against routine laboratory evaluations, including fat-soluble vitamin testing, liver function testing, glucose monitoring, and blood counts for people with CRMS/CFSPID (Grade D).

Measuring FE at the initial assessment is important to evaluate for exocrine pancreatic function because pancreatic insufficiency is a clinical feature of CF. FE levels can fluctuate in the first year of life; therefore, a single abnormal FE level should be interpreted cautiously, and repeat testing may be warranted^43^ if symptoms of steatorrhea or failure to thrive develop. Most people with CRMS/CFSPID exhibit normal growth and nutrition and are pancreatic sufficient; thus, the presence of pancreatic insufficiency would strongly support reclassification to CF.^35,44,45^

There is no evidence that laboratory results, such as electrolyte concentrations or liver function tests, are abnormal in people with CRMS/CFSPID.^35^ Excessive testing leads to overmedicalization and increased cost of care. Therefore, laboratory tests should only be considered when clinically indicated.

#### Pulmonary Function Testing

- The CFF recommends against routine pulmonary function testing (PFT; ie, spirometry, multiple-breath washout) for people with CRMS/CFSPID (Grade D).

Spirometry and multiple-breath washout are normal in people with CRMS/CFSPID^4,33,35^ and do not affect reclassification to CF. PFTs should be considered if clinical concern for respiratory disease arises, and if abnormal, may support reclassification to CF.

#### Radiographic Imaging

- The CFF recommends against routine chest radiographs for people with CRMS/CFSPID (Grade D).

Chest imaging is normal in people with CRMS/CFSPID within the first years of life^35^ and does not inform reclassification to CF. Chest radiographs should be considered if clinical concerns arise.

### Treatment

#### Infection Prevention and Control

- The CFF recommends, for people with CRMS/CFSPID, the implementation of standard CF infection prevention and control (IPC) guidelines in health care settings and situations in which there is a high likelihood of being in close contact with multiple pwCF or people with CRMS/CFSPID (Grade B).

The acquisition of *Pa* may guide reclassification to CF, and there is a risk that exposure to pathogens commonly associated with CF may negatively impact the individual.^46^ This risk is most substantial in the health care setting in which close contact could occur among multiple pwCF or people with CRMS/CFSPID. IPC guidelines have been adopted by CF programs with minimal negative impact.^47^ The implementation of IPC guidelines in non-health care settings, such as schools, may have significant negative psychological effects that outweigh potential benefits and are not recommended.

#### Infectious Disease Interventions

- The CFF recommends, for people with CRMS/CFSPID, selectively offering inhaled antibiotics for the treatment of *Pa* based on a positive respiratory culture (Grade C).
- The CFF recommends, for people with CRMS/CFSPID and an unexplained prolonged cough (>2 weeks), selectively offering the use of oral antibiotics (Grade C).

A higher prevalence of *Pa* is reported in respiratory cultures from people with CRMS/CFSPID compared with those collected from children without CF,^2,4,41,42^ and treatment to eradicate incident *Pa* infection is recommended for pwCF.^48^ However, the clinical impact of *Pa* in respiratory cultures from people with CRMS/CFSPID is unclear. *Pa* may clear spontaneously, (ie, independent of whether treatment is provided),^49^ calling into question the value of *Pa* eradication therapy. The committee recommends selectively offering this treatment after a SDM process incorporating the potential benefits, risks, and treatment burden of inhaled antibiotic therapy in people with CRMS/CFSPID with positive respiratory culture for *Pa*. If the decision is to treat, then follow-up cultures are warranted to ensure *Pa* clearance.

Precedent is established regarding use of oral antibiotics for prolonged cough for individuals with respiratory conditions other than CF, including tracheobronchomalacia, protracted bacterial bronchitis, non-CF bronchiectasis and neuromuscular disease.^50–54^ The committee discussed this consideration while weighing the potential benefits and harms of antibiotic therapy. The use of any antibiotic for eradication or treatment may depend on clinical status of the individual, and the risks and benefits of treatment burden should be assessed for each family. For people with CRMS/CFSPID with a cough lasting >2 weeks, other etiologies of persistent cough, such as asthma, should be considered.

#### Nutritional Interventions

- The CFF recommends, for people with CRMS/CFSPID with adequate growth, that nutritional management be provided under the direction of the PCP (Grade B).
- The CFF recommends, for people with CRMS/CFSPID with a downward trajectory of weight-for-age percentile or z-score (eg, crossing percentiles), that screening and evaluation be provided by a dietitian with experience in the management of CRMS/CFSPID and CF (Grade B).
- The CFF recommends against salt supplementation for people with CRMS/CFSPID (Grade D).
- The CFF recommends against the use of fat-soluble vitamins for people with CRMS/CFSPID (Grade D).

No evidence exists to support the use of supplemental nutrition, caloric modifications, or CF-specific diets for people with CRMS/CFSPID who are exhibiting normal growth.^55^ A downward growth trajectory, as measured by weight-for-age percentile or weight z-score, may be a sign of reclassification to CF. Children who demonstrate this pattern should undergo nutritional screening and appropriate intervention by dietitians with experience in CRMS/CFSPID and CF. Given that people with CRMS/CFSPID have intermediate or normal sweat [Cl^−^], excessive salt loss is not expected, and potentially harmful consequences could be conferred by a high-salt diet.^56,57^ No evidence suggests the need for supplementing fat-soluble vitamins to people with CRMS/CFSPID^35^ because deficits in these vitamins have not been observed, and routine laboratory tests are not being monitored.

#### Pulmonary Interventions

- The CFF recommends against the routine use of airway clearance therapy (ACT) for people with CRMS/CFSPID (Grade D).
- The CFF recommends for people with CRMS/CFSPID experiencing new respiratory symptoms selectively offering the use of ACT (Grade C).
- The CRMS/CFSPID guideline committee recommends against the use of CFTR modulators for people with CRMS/CFSPID (Grade D).
- The CFF has determined that there is insufficient evidence to recommend for or against the use of medications usually used to treat CF respiratory symptoms for people with CRMS/CFSPID (Grade I).

Routine ACT and its effects on outcomes, such as bronchiectasis, are unclear for people with CRMS/CFSPID; hence, daily use is not recommended. ACT has been recommended for the management of acute respiratory symptoms for pediatric patients with other pulmonary conditions^58,59^; thus, for people with CRMS/CFSPID and new respiratory symptoms, ACT may be considered. Initiation should be discussed with the family because ACT may add a significant burden. The use of cost-effective methods (such as manual percussion) should be attempted.

No studies have been reported in which the authors investigated the outcomes of CFTR modulator therapies among people with CRMS/CFSPID, and potential adverse effects are associated with these medications.^60–62^ If people with CRMS/CFSPID develop signs and symptoms warranting the use of CFTR modulators, reclassification to CF may be justified.

Medications that are commonly used for CF lung disease,^63,64^ (ie, dornase alfa, hypertonic saline, and low-dose azithromycin) have not been evaluated in people with CRMS/CFSPID. The potential benefits of these medications for people with CRMS/CFSPID are unclear because the degree of CFTR dysfunction in CRMS/CFSPID may not necessarily result in significant changes in airway surface liquid. Weighed against the risks of medical complexity, treatment burden, and cost, there is insufficient evidence to either recommend for or against use of these medications. For people with CRMS/CFSPID with chronic respiratory symptoms that would require consideration of therapy, the reassessment of CF is warranted.

### Communication and Psychosocial Issues

#### Communication With Families

- The CFF recommends, for people with CRMS/CFSPID, that HCPs assess and consider social determinants of health (SDOH) that can influence the understanding and psychological impact of a CRMS/CFSPID diagnosis and tailor communications appropriately (Grade B).
- The CFF recommends, for people with CRMS/CFSPID, that HCP tailor communication about *CFTR* variants based on SDM to minimize psychological, cognitive, and other barriers to processing and understanding genetic information (Grade B).
- The CFF recommends, for people with CRMS/CFSPID, that clear, concise, consistent, and timely information about the uncertainty related to the CRMS/CFSPID diagnosis is provided using family-centered communication strategies (Grade B).
- The CFF recommends, for people with CRMS/CFSPID and their families, that gradual, clear, and consistent, verbal and written, developmentally appropriate education about CRMS/CFSPID is provided at diagnosis, at follow-up visits, and at the time of reproductive decision-making (Grade B).

The psychological impact of the uncertainty associated with CRMS/CFSPD is challenging for parents and highlights the need for consistent and clear communication between families, patients, and HCP.^65,66^ SDOH can influence a persons’ understanding of a diagnosis based on previous knowledge, emotional state, genetic and health literacy, and perceptions of test results.^66–69^ Screening for SDOH will identify social needs for which CFCC can provide links to further services. Parental learning preferences should be considered. Qualified medical language interpreters should be involved unless the provider is certified as a proficient interpreter in the primary language of the people with CRMS/CFSPID and their caregivers.

Perceived lack of knowledge of the person communicating NBS results has been linked to parental distress.^70^ Clear, concise, and consistent information with plans for future follow-up is necessary for parents managing the uncertainty associated with the diagnosis of CRMS/CFSPID.^2,65^ Accurate information, with reassurance and without conjecture, is critical for moderating parental response to this diagnosis.^66,71^ Educational tools have been developed to assist HCP when communicating information to families the uncertainty of other diagnoses.^70^ Additional information on this topic is available in the Supplemental Information.

#### Communication With Primary Care

- The CFF recommends, for people with CRMS/CFSPID, that the PCPs and other HCPs involved in the care of individuals with CRMS/CFSPID receive accurate and up-to-date education about CRMS/CFSPID, its management, and the state’s NBS program (Grade B).

When communicating with parents and HCPs, it is important to provide information regarding further symptoms that may arise and when to refer to a CFCC. Because NBS algorithms vary by state,^1^ CFCCs should serve as a resource with current information for PCPs to help educate and clarify any *CFTR* results, SCT, or other evaluations that pertain to CRMS/CFSPID.^3,6^

#### Screening for Depression and Anxiety

- The CFF recommends that at least 1 primary caregiver of people with CRMS/CFSPID be offered screening for depression and anxiety annually (Grade B).
- The CFF recommends, for people with CRMS/CFSPID aged 12 years and older still being followed by CFCC, that screening for depression and anxiety be provided annually (Grade B).

The International Committee on Mental Health in Cystic Fibrosis^72^ and the USPSTF screening guidelines^73,74^ recommend annual screening for depression and anxiety in people aged 12 years and older. To be consistent with guidelines, the committee recommends utilizing the Patient Health Questionnaire-9 and Generalized Anxiety Disorder-7 forms. A team member with expertise and training in mental health should be identified to implement screening, follow-up, and referral.^72^ Although there is insufficient evidence to recommend routine screening for anxiety and depression in people with CRMS/CFSPID aged 7 to 11 years, this population should be clinically evaluated for these problems when significant symptoms or behavioral concerns are reported or if caregiver depression or anxiety scores are elevated.

Mothers of people with CRMS/CFSPID exhibit increased rates of anxiety, depression, and postpartum depression that are comparable to rates detected among mothers of pwCF.^75^ News of the CRMS/CFSPID designation may create a state of cognitive uncertainty regarding the nature of the diagnosis and prognosis. This mental state contributes to clinically significant distress for parents and caregivers^76–78^ like that experienced by parents of pwCF.^66^ Discussions should be family-centered and may include asking about the caregiver’s emotional and mental health concerns and SDOH, racism, poverty, and relational health.^73,79,80^

### No Consensus

The committee was unable to reach a consensus of 80%, with 20 voting for recommendation (74%) and 7 voting against a recommendation for selectively offering chest computed tomography (CT) scans. There are few reports of chest CT imaging in people with CRMS/CFSPID,^35,49,81,82^ and abnormal findings are rare. Many members of the committee felt that chest CTs are not routinely recommended for pwCF and should not be recommended for people with CRMS/CFSPID.

## LIMITATIONS AND AREAS FOR FUTURE RESEARCH

Although numerous studies of CRMS/CFSPID have been published since 2009, most encompass single-center analyses with relatively small cohorts. The CFF Patient Registry (CFFPR) contains many people with CRMS/CFSPID and has been used to study CRMS outcomes. However, there is no requirement for inclusion in the CFFPR, and many people with CRMS/CFSPID are not represented in this database. No randomized clinical trials of people with CRMS/CFSPID have been reported, and it is unlikely that such future studies will be performed, all of which affect the strength of the present recommendations. Enrolling more people with CRMS/CFSPID into CFFPR will be a primary way to develop larger multicenter cohort studies and better guide management.

Genetic testing technology is continually advancing. Tests with limited availability now (eg, compete sequencing of the intronic regions of *CFTR*) may become more widely available in the future. Establishing the disease liability of VVCCs and variants of uncertain significance represents an unmet need that may help assess the risk of reclassifying CRMS/CFSPID to CF, as well as guide the selection of *CFTR* variants in NBS algorithms. Knowledge of state NBS algorithms and access to further genetic testing will be essential because these platforms continue to vary across the United States.^1^ People with *CFTR*-related disorder have *CFTR* and SCT results similar to people with CRMS/CFSPID,^83^ but the likelihood of people with CRMS/CFSPID developing into *CFTR*-related disorder is currently unknown. Pursuing this area of research is also an important area for future research.

Working closely with PCPs is a mainstay of care for people with CRMS/CFSPID. Table 4 is a suggestion for the continued monitoring and care of people with CRMS/CFSPID and can be employed at both specialty care and primary care. Determining an appropriate time to discharge from care at a specialized center will require SDM with the people with CRMS/CFSPID, family, specialty providers and PCPs. Guidance regarding when to refer back to specialty clinics (eg, change in respiratory symptoms) needs to be developed.

We present this evidence-based guideline for the management of CRMS. Most of the recommendations were grade C because of the limited data that are currently available. As more people with CRMS/CFSPID are followed for longer periods of time, reassessment of these recommendations will be required. Additionally, research is needed to assess clinical benefits of treating pulmonary symptoms with medications commonly used for CF lung disease, antibiotic therapy for *Pa* in respiratory cultures, and the use of PFTs or chest imaging for monitoring development of lung disease in people with CRMS/CFSPID.

## Supplementary Material

Supplemental Information

## Figures and Tables

**TABLE 1:** USPSTF Guideline Recommendations^8^

Grade	Definition	Suggestions for Practice
A	The USPSTF recommends the service. There is high certainty that the net benefit is substantial.	Offer or provide this service.
B	The USPSTF recommends the service. There is high certainty that the net benefit is moderate or there is moderate certainty that the net benefit is moderate to substantial.	Offer or provide this service.
C	The USPSTF recommends selectively offering or providing this service to individual patients based on professional judgment and patient preferences. There is at least moderate certainty that the net benefit is small.	Offer or provide this service for selected patients, depending on individual circumstances.
D	The USPSTF recommends against the service. There is moderate or high certainty that the service has no net benefit or that the harms outweigh the benefits.	Discourage the use of this service.
I	The USPSTF concludes that the current evidence is insufficient to assess the balance of benefits and harms of the service. Evidence is lacking, of poor quality, or conflicting, and the balance of benefits and harms cannot be determined.	Read the clinical considerations section of the USPSTF Recommendation Statement. If the service is offered, patients should understand the uncertainty about the balance of benefits and harms.

**TABLE 2:** Consensus Recommendation Statements: 30 Statements That Made Consensus

	Statement	Grade	Certainty	Percentage Agreement
Genetics				
1	The CFF recommends that people with CRMS/CFSPID who have <2 disease-causing variants identified by NBS, should undergo sequencing of the coding and flanking regions and del/dup analysis of the coding and exon flanking regions of *CFTR*.	B	Moderate	100%
2	The CFF recommends for people with CRMS/CFSPID selectively offering full-gene *CFTR* sequencing including intronic regions when the *CFTR* genotype remains incomplete after coding and flanking region sequencing and del/dup.	C	Low	100%
3	The CFF recommends *CFTR* genetic evaluation for parents of people with CRMS/CFSPID when phasing of the *CFTR* variants (ie, in *cis* or *trans*) would inform the diagnostic status of the individual by confirming the inheritance pattern.	A	High	100%
4	The CFF recommends offering *CFTR* genetic evaluation for siblings of people with CRMS/CFSPID.	B	Moderate	100%
5	The CFF recommends for families of people with CRMS/CFSPID that HCPs providing genetic counseling should have training and/or clinical expertise in CF and genetics. A licensed or certified GC should be accessible to families of people with CRMS/CFSPID for further support, including discussions regarding future reproductive decision-making.	B	Moderate	96.2%
Monitoring				
6	The CFF recommends for people with CRMS/CFSPID to receive at least annual follow-up by a CF clinician and nurse, with an initial assessment to include social work and/or MHC and/or genetic counseling provider. Continued follow-up by social work, MHC and genetic counseling should be part of the care of CRMS/CFSPID, depending on the needs of that individual and family.	B	Moderate	96.2%
7	The CFF recommends for people with CRMS/CFSPID repeating sweat chloride testing at 6 months of life and annually at least until age 8 y.	B	Moderate	100%
8	The CFF recommends for people with CRMS/CFSPID selectively offering cystic fibrosis respiratory cultures at each visit (at least until age 8 y) and as clinically indicated for respiratory symptoms.	C	Moderate	96.2%
9	The CFF recommends for people with CRMS/CFSPID that measurement of FE be provided at the initial assessment. Further testing of FE can be provided when clinically appropriate.	B	High	100%
10	The CFF recommends against routine laboratory evaluations, including fat-soluble vitamin testing, liver function testing, glucose monitoring, and blood counts for people with CRMS/CFSPID.	D	Moderate	100%
11	The CFF recommends against routine PFT (ie, spirometry, multiple breath washout) for people with CRMS/CFSPID.	D	Moderate	88.5%
12	The CFF recommends against routine chest radiographs for people with CRMS/CFSPID.	D	Moderate	92.3%
Treatment				
13	The CFF recommends for people with CRMS/CFSPID implementation of standard CF IPC guidelines in health care settings and situations in which there is a high likelihood of being in close contact with multiple pwCF or CRMS/CFSPID	B	Moderate	92.3%
14	The CFF recommends for people with CRMS/CFSPID selectively offering inhaled antibiotics for the treatment of *Pa* based on a positive respiratory culture.	C	Low	96.2%
15	The CFF recommends for people with CRMS/CFSPID and unexplained prolonged cough (>2 wk) selectively offering the use of oral antibiotics.	C	Low	96.2%
16	The CFF recommends for people with CRMS/CFSPID and adequate growth that nutritional management be provided under the direction of the PCP.	B	High	100%
17	The CFF recommends for people with CRMS/CFSPID and a downward trajectory of wt for age percentile or z-score (eg, crossing percentiles) that screening and evaluation be provided by a dietitian with experience in the management of CRMS/CFSPID and CF.	B	High	100%
18	The CFF recommends against salt supplementation for people with CRMS/CFSPID.	D	Moderate	100%
19	The CFF recommends against the use of fat-soluble vitamins for people with CRMS/CFSPID.	D	Moderate	100%
20	The CFF recommends against the routine use of airway clearance for people with CRMS/CFSPID.	D	Low	100%
21	The CFF recommends for people with CRMS/CFSPID and experiencing new respiratory symptoms, selectively offering the use of airway clearance.	C	Low	100%
22	The CRMS/CFSPID guidelines committee recommends against the use of CFTR modulators for people with CRMS/CFSPID.	D	Moderate	94.4%
23	The CFF recommends that there is insufficient evidence to recommend for or against the use of medications usually used to treat CF respiratory symptoms for people with CRMS/CFSPID	I		80.8%
Psychosocial/communication				
24	The CFF recommends for people with CRMS/CFSPID that HCPs assess and consider SDOH that can influence the understanding and psychological impact of a CRMS/CFSPID diagnosis and tailor communications appropriately.	B	Moderate	100%
25	The CFF recommends for people with CRMS/CFSPID that HCPs tailor communication about *CFTR* variants based on SDM to minimize psychological, cognitive, and other barriers to processing and understanding genetic information.	B	High	100%
26	The CFF recommends for people with CRMS/CFSPID that clear, concise, consistent, and timely information about the uncertainty related to the CRMS/CFSPID diagnosis is provided using family-centered communication strategies.	B	Moderate	96.2%
27	The CFF recommends for people with CRMS/CFSPID and their families that gradual, clear, and consistent verbal and written developmentally appropriate education about CRMS/CFSPID is provided at diagnosis, at follow-up visits and at the time of reproductive decision-making.	B	Moderate	100%
28	The CFF recommends for people with CRMS/CFSPID that the PCP and other HCPs involved in the care of individuals with CRMS/CFSPID receive accurate and up-to-date education about CRMS/CFSPID, its management, and their state’s NBS program.	B	Moderate	96.2%
29	The CFF recommends for at least 1 primary caregiver of people with CRMS/CFSPID offering screening for depression and anxiety annually.	B	Moderate	96.2%
30	The CFF recommends for people with CRMS/CFSPID age 12 y and older still being followed by CF care center that screening for depression and anxiety be provided annually.	B	Moderate	96.2%

**TABLE 3:** Non-Consensus Recommendation Statement: 1 Statement That Did Not Make Consensus

PICO	Statement	Grade	Certainty	Percentage Agreement
Should individuals with CRMS/CFSPID have chest CT imaging?	Consensus was not achieved on the statement recommending selectively offering chest CT scans to people with CRMS/CFSPID	C	Low	74.1%

**TABLE 4:** Monitoring and Care for the Person With CRMS/CFSPID

Age at Visit	Day of First Sweat Test	6 mo	1–8 y	9–11 y^a^	12 y and older^a^
Intervention					
Discuss diagnosis	P	P	A	A	A
Sweat test	P	P	A	C	C
FE	P	C	C	C	C
Cystic fibrosis culture	—	C	C	C	C
Assessment of variant classification in national database (eg, CFTR2)	P	P	A	A	A
Sequencing (if not done at birth)	C	C	C	C	C
Del/dup testing	C	C	C	C	C
Infection control	P	P	A	A	A
CF patient registry consent	—	P	C	C	C
Genetic counseling	P	P	C	C	C
Mental health screening of parents	P	P	A	A	A
Mental health screening of patient	—	—	C	C	A

A, annually perform; C, consider; P, perform. —, not needed at this age.

a If still being followed at CFCC.

## ABBREVIATIONS

ACT: airway clearance therapy
CF: cystic fibrosis
CFCC: cystic fibrosis care centers
CFF: Cystic Fibrosis Foundation
CFFPR: CFF Patient Registry
CFSPID: cystic fibrosis screen-positive, inconclusive diagnosis
*CFTR*: cystic fibrosis transmembrane conductance regulator
CRMS: CFTR-related metabolic syndrome
CT: computed tomography
del/dup: deletion/duplication
ECFS: European Cystic Fibrosis Society
FE: fecal elastase
GC: genetic counselor
HCP: health care professional
IPC: infection prevention and control
MHC: mental health coordinator
NBS: newborn screening
*Pa*: *Pseudomonas aeruginosa*
PCP: primary care provider
PFT: pulmonary function testing
PICO: patient, intervention, comparison, outcome
pwCF: people with cystic fibrosis
SDM: shared decision-making
SDOH: social determinants of health
sweat [Cl^−^]: sweat chloride concentration
USPSTF: US Preventive Services Task Force
VVCCs: variants of varying clinical consequence
