# Supplementary material for: Cystic Fibrosis Foundation Evidence-Based Guideline for the Management of CRMS/CFSPID
Source: Pediatrics. Author manuscript; Available in PMC 2025 Jan 30. (PMC11781860; doi:10.1542/peds.2023-064657)
Supplement: Supplemental Information [file NIHMS2047495-supplement-Supplemental_Information.pdf]

Supplemental Information

GENETICS

Genetic testing has evolved over the years and continues to evolve. It is important to clarify the distinction between "full *CFTR* sequencing," which is an unofficial term that has been used to distinguish between a *CFTR* test that involves a limited variant panel, and sequencing of the coding and flanking regions. The latter was and remains a more comprehensive test that has the ability to identify nearly any variant in the exons and flanking intronic regions, rather than only specific variants from a panel. However, the recent availability of gene sequencing that includes both exons and introns has caused considerable confusion with the unofficial term of "full *CFTR* sequencing."

Gene sequencing involving all exons and flanking intronic regions of *CFTR* is now recommended for all pwCRMS who do not have 2 identified causal *CFTR* variants after NBS. Generally speaking, this is a test that is widely available in most molecular genetics laboratories that include *CFTR* in their test offerings. In many individuals, *CFTR* sequencing of the coding and flanking intronic regions will identify a second variant; this can provide additional prognostic information for an infant and improve the accuracy of risk estimates for the infant's parents' future reproductive risk. Until recently, this type of testing was frequently referred to as "full *CFTR* sequencing," but it should be noted that it typically does not delve deep into the intronic regions.

Most diagnostic *CFTR* sequencing tests that include the coding and flanking regions are performed using next-generation sequencing technology, also called next-gen sequencing or NGS. Although NGS-based tests reliably identify small genetic changes involving 1 or a few nucleotides, which compromise most of the known *CFTR* variants, they are more limited in their ability to detect large structural variants such as exon deletions or duplications (eg, CFTRdele2,3, which is a deletion of exons 2 and 3 of *CFTR*). Some laboratories will perform a separate analysis of the NGS results to identify such variants, but others will not or may require that deletion and duplication testing be ordered separately. This will vary by laboratory and by state if a particular state's NBS protocol includes a *CFTR* sequencing tier. In the context of pwCRMS and only 1 identified *CFTR* variant, it is important to determine if deletion and duplication analysis has been performed (either as part of NBS or as part of an additional *CFTR* sequencing test ordered in the clinic) so that a decision can be made regarding further genetic testing. GCs or other laboratory staff should be consulted for help interpreting genetic test results or for assistance in determining the extent of testing performed.

A new recommendation is the selective offering of additional *CFTR* testing that involves sequencing of the entirety of the coding and noncoding (intronic, upstream, and downstream) regions. At the time of the writing of this article, there is limited availability of *CFTR* testing that includes all noncoding regions. This is expected to change over time. Although there is no official or accepted term, "full-gene *CFTR* sequencing" is sometimes used, or it can be specified that this is a test that includes the noncoding regions of *CFTR*.

Offering additional *CFTR* genetic testing that includes intronic regions is most appropriate to consider in infants who have 1 causal *CFTR* variant identified and a heightened suspicion that they may meet criteria for a CF diagnosis in the future. This may include infants with a sweat [Cl$^-$] closer to the diagnostic threshold of 60 mmol/L (rather than closer to the normal range of <30 mmol/L) or infants whose sweat [Cl$^-$] is increasing over multiple tests. Another scenario to consider for *CFTR* sequencing that includes intronic regions is infants who have persistently elevated, but nondiagnostic, sweat chlorides and whose single identified variant is known to be associated with mild or varying clinical consequences, such as R117H;7T (c.350G>A with the 7T variant in the intron 9) or D1152H (c.354G>C), and no second variant identified after coding and flanking region sequencing and del/dup analysis.

It is also important to consider the extent of the *CFTR* testing performed as part of or after NBS. If sequencing the coding and flanking intronic regions included many basepairs of flanking intronic region (such as >50 basepairs) or several deep intronic variants (eg, 1811 + 1.6kbA>G [c.1680-886A>G]), then a test that includes sequencing of all noncoding regions may not provide a significant increase in detection rate. However, if a sequencing test is limited to a smaller number of intronic basepairs (eg, <15) and a small number of deep intronic variants, then a test that includes sequencing of the entire noncoding region may be more likely to identify a second variant. Again, consultation with a GC knowledgeable in CF genetics or laboratory staff may be helpful in ascertaining the extent to which noncoding regions are being sequenced.

It is important for clinicians to note that variants in the noncoding regions of *CFTR* may be interpreted by testing laboratories as variants of uncertain significance because this region is considerably less well-studied than the coding region. A discussion of the family's tolerance for uncertainty may aid in determining who should undergo this additional testing.

We are including Supplemental Table 5 as a genetic glossary of terms to better understand these concepts.

COMMUNICATION AND PSYCHOSOCIAL ISSUES

The diagnosis of CRMS/CFSPID is challenging for parents, caregivers, and providers. Variability exists in the communication of results and expected outcomes. It is not a lack of knowledge of the results but the exact trajectory the individual with CRMS/CFSPID will have. Presenting individualized written

information with genetic counseling can improve parent understanding of genetic results and implications. Awareness of the environment can influence understanding and retention of educational information.[68,84] A 4-step model for communicating diagnostic uncertainty may improve the conveyance of the news by promoting conversations while decreasing the possibility of misinterpretation.[77] Follow-up educational sessions during subsequent visits may provide parental appreciation with further benefit to clarify information while addressing additional questions.[71,85] Counseling approaches that decreased parental distress included matching the timing and amount of information to the parents' needs, showing empathy to parents' distress, instilling a sense of hope, personalizing the counseling approach, and offering acts of hospitality. The same approach may not suit all parents.[69]

Educational tools have been developed, including condition-specific communication checklists, as well as e-mail and letter templates for healthcare providers to provide information to families and assist healthcare providers in addressing the uncertainty surrounding NBS,[70] although this is not specific to CRMS/CFSPID. Using teach-back techniques will ensure that educational information about CRMS/CFSPID is understood.[68] Delivering health information in a distraction-free environment, in person, with factual data, without jargon using varied communication channels (written, verbal and video) in the family's primary language will improve understanding and retention.[85,86] Multiple opportunities should be provided to educate parents including follow-up phone calls after communication of the diagnosis of CRMS/CFSPID.[68] Additional educational encounters will provide opportunities to review previous discussions, answer questions, and clarify information. PCPs should be informed on when to seek advice for symptoms which could present over time and at different developmental stages.[3,6]

The explanation of complicated genetic terminology can be difficult for parents of an infant with CRMS/SFSPID to understand. Having access to a GC for the discussion of the genetic findings and psychosocial team members to provide support will promote understanding of the diagnosis.[3,6,27] Parents who received genetic counseling at the time of their infant's diagnostic sweat test had significantly higher long-term retention of genetic knowledge than those without genetic counseling.[31] Genetic counseling at regular intervals allows for timely, accurate, supportive, and nondirective information on recurrence risk and reproductive options.[29]

METHODS

The CFF invited a multidisciplinary team including physicians, GCs, a dietician, a social worker, a nurse, an NBS coordinator, and the parents of children with, or who had once been diagnosed with, CRMS/CFSPID to participate in the development of these consensus guidelines. Committee members were identified by the chairs, the CF Foundation staff, and a discipline-specific call for applications. The discipline-specific call for applications was distributed over the CF listservs. All committee members adhered to the CF Foundation's conflict of interest policy and disclosed any potential conflicts before the initiation of the guideline. The committee met for their first meeting on October 1, 2021 to determine the scope of the work and divide into 4 workgroups. The workgroups focused on the psychosocial aspects of the diagnosis, genetic workup for the diagnosis, and the monitoring and treatment of the diagnosis. Several PICO questions were developed by each workgroup. CFF staff and Deanna Green, MD, MHS, performed literature searches in PubMed and Cumulated Index to Nursing and Allied Health Literature (CINAHL) with search terms specific to the topics, and workgroup members provided advice throughout the process.

This search resulted in 773 articles found across all PICO questions. After reviewing the literature, 721 articles were excluded: 421 articles were excluded at the title level, 221 were excluded at the abstract level, and 79 were excluded at the full-text level. The workgroups conducted a single review of the literature at the title and abstract levels and a double review at the full-text level. Articles for which there was no agreement during the individual review of the full text were discussed by the reviewers to come to agreement on whether to include the article.

For all workgroups, articles were excluded for the following reasons: descriptive or review or editorial papers, does not relate to the question, article did not relate to CRMS/CFSPID, case reports, and basic science.

The workgroups developed draft recommendations on the basis of the results of the literature search. The committee discussed the workgroup recommendations on monthly phone calls. The committee reconvened on October 6, 2022 to revise and adopt the draft recommendation statements developed in the workgroups. The committee established an a priori voting threshold of 80% agreement.

The manuscript was reviewed by the entire committee before distribution for public comment on May 30, 2023. Public comment was distributed to the CF community using the CFF's medical list servs (these listservs included disciplines both represented and not represented on the committee), shared with the ECFS and CF Canada, and Community Voice (https://www.cff.org/get-involved/community-voice), a platform for individuals with CF and their family members to provide feedback on the CFF's initiatives. During this time, the literature search was rerun to ensure that articles published since the initial literature search were captured and relevant articles were reviewed.

The committee reviewed and acknowledged or addressed each of the comments received during public comment.

After the revised statements were approved by the committee, the text was revised to reflect these changes. The revised manuscript was then sent to the committee for final approval before submission for publication.

SUPPLEMENTAL TABLE 5 Literature Searches								
PICO	Group	Question	Search (PubMed)	Date	Yield	Search (CINAHL)	Date	Yield
1	Psychosocial	Should communication surrounding the uncertainty related to the CRMS diagnosis include *CFTR* mutation-specific information?	((((((((“CRMS”) OR (“CFTR Related Metabolic Syndrome”)) OR (“CFSPID”)) OR (“CF, Screen Positive Inconclusive Diagnosis”)) OR (“cystic fibrosis transmembrane conductance regulator/ genetics*”[All Fields]))) AND ((((“infant”[All Fields]) OR (“newborn”[All Fields])) OR (“neonatal screening”[All Fields])) OR (“newborn screening”[All Fields]))) AND ((((((((diagnosis) OR (CFTR mutation)) OR (uncertainty)) OR (communication)) OR (risk communication)) OR (vulnerable child)) OR (health communication)))	3/29/2022	725	(“cystic fibrosis transmembrane conductance regulator - related metabolic syndrome” or “cystic fibrosis screen positive inconclusive diagnosis” or “CRMS” or “CFSPID” or “CFTR - related metabolic syndrome” or “cystic fibrosis transmembrane conductance regulator/genetic*” or “CFTR genetic*”) AND (diagnosis or “CFTR mutation” or uncertainty or communication or “risk communication” or vulnerable child” or “health communication”) excluded medline	3/19/22	10
2	Psychosocial	What is the most effective communication strategy to convey the uncertainty related to the CRMS diagnosis?	(((diagnosis) OR (uncertainty)) OR (communication)) AND ((((((((“CRMS”) OR (“CFTR Related Metabolic Syndrome”)) OR (“CFSPID”)) OR (“CF, Screen Positive Inconclusive Diagnosis”)) OR (“cystic fibrosis transmembrane conductance regulator/ genetics*”[All Fields]))) AND ((((“infant”[All Fields]) OR (“newborn”[All Fields])) OR (“neonatal screening”[All Fields])) OR (“newborn screening”[All Fields])))	3/29/2022	590	(“cystic fibrosis transmembrane conductance regulator - related metabolic syndrome” or “cystic fibrosis screen positive inconclusive diagnosis” or “CRMS” or “CFSPID” or “CFTR - related metabolic syndrome” or “cystic fibrosis transmembrane conductance regulator/genetic*” or “CFTR genetic*”) AND (diagnosis or “CFTR mutation” or uncertainty or communication or “risk communication” or vulnerable child” or “health communication”) excluded medline	3/19/22	10
3	Psychosocial	Should CF health care providers screen parent/care providers of children with CRMS and adolescents of CRMS from depression and anxiety?	((((((((“CRMS”) OR (“CFTR Related Metabolic Syndrome”)) OR (“CFSPID”)) OR (“CF, Screen Positive Inconclusive Diagnosis”)) OR (“cystic fibrosis transmembrane conductance regulator/ genetics*”[All Fields]))) AND ((((“infant”[All Fields]) OR (“newborn”[All Fields])) OR (“neonatal screening”[All Fields])) OR (“newborn screening”[All Fields]))) AND (((depression) OR (anxiety)) OR (mental health))	3/29/2022	12	(“cystic fibrosis transmembrane conductance regulator - related metabolic syndrome” or “cystic fibrosis screen positive inconclusive diagnosis” or “CRMS” or “CFSPID” or “CFTR - related metabolic syndrome” or “cystic fibrosis transmembrane conductance regulator/genetic*” or “CFTR genetic*”) AND (depression or anxiety or “mental health”) excluded medline	3/19/22	2

SUPPLEMENTAL TABLE 5 Continued

PICO	Group	Question	Search (PubMed)	Date	Yield	Search (CINAHL)	Date	Yield
4	Psychosocial	How should/to what degree should children, adolescents and the community receive specific education about the diagnosis of CRMS? Including information on follow-up care	(((((((“CRMS”) OR (“CFTR Related Metabolic Syndrome”)) OR (“CFSPID”)) OR (“CF, Screen Positive Inconclusive Diagnosis”)) OR (“cystic fibrosis transmembrane conductance regulator/ genetics*”[All Fields]))) AND ((((“infant”[All Fields]) OR (“newborn”[All Fields])) OR (“neonatal screening”[All Fields])) OR (“newborn screening”[All Fields]))) AND ((diagnosis) AND (education))	3/29/2022	22	(“cystic fibrosis transmembrane conductance regulator - related metabolic syndrome” or “cystic fibrosis screen positive inconclusive diagnosis” or “CRMS” or “CFSPID” or “CFTR - related metabolic syndrome” or “cystic fibrosis transmembrane conductance regulator/genetic*” or “CFTR genetic*”) AND (education or diagnosis or “CF education” or “child health education” or “adolescent health education” or “vulnerable child”) excluded medline	3/19/22	8
5	Psychosocial	What disparities in communication can be addressed surrounding the diagnosis of CRMS?	(((((((“CRMS”) OR (“CFTR Related Metabolic Syndrome”)) OR (“CFSPID”)) OR (“CF, Screen Positive Inconclusive Diagnosis”)) OR (“cystic fibrosis transmembrane conductance regulator/ genetics*”[All Fields]))) AND ((((“infant”[All Fields]) OR (“newborn”[All Fields])) OR (“neonatal screening”[All Fields])) OR (“newborn screening”[All Fields]))) AND ((healthcare disparities) OR (communication))	3/29/2022	18	(“cystic fibrosis transmembrane conductance regulator - related metabolic syndrome” or “cystic fibrosis screen positive inconclusive diagnosis” or “CRMS” or “CFSPID” or “CFTR - related metabolic syndrome” or “cystic fibrosis transmembrane conductance regulator/genetic*” or “CFTR genetic*”) AND (“healthcare disparities” or communication or “diagnosis communication” or disparity in communication” or demographics or underrepresented or “language barriers” or knowledge or understanding or “vulnerable child” or “primary care” or insurance or “cultural diversity”) excluded medline	3/19/22	0
6	Genetics	What genetic testing should be performed on an infant with CRMS/CFSPID and <2 *CFTR* variants identified by variant panel?	(((((((“CRMS”) OR (“CFTR Related Metabolic Syndrome”)) OR (“CFSPID”)) OR (“CF, Screen Positive Inconclusive Diagnosis”)) OR (“cystic fibrosis transmembrane conductance regulator/ genetics*”[All Fields]))) AND ((((“infant”[All Fields]) OR (“newborn”[All Fields])) OR (“neonatal screening”[All Fields])) OR (“newborn screening”[All Fields]))) AND ((((genetic testing)) OR (genetic variant)) OR (variant panel))	3/29/2022	281	(“cystic fibrosis transmembrane conductance regulator - related metabolic syndrome” or “cystic fibrosis screen positive inconclusive diagnosis” or “CRMS” or “CFSPID” or “CFTR - related metabolic syndrome” or “cystic fibrosis transmembrane conductance regulator/genetic*” or “CFTR genetic*”) AND (“genetic testing” or “genetic screening” or “genomic sequencing” or “genome sequencing” or genome mapping” or infant or newborn) excluded medline	3/19/22	6

SUPPLEMENTAL TABLE 5 Continued

PICO	Group	Question	Search (PubMed)	Date	Yield	Search (CINAHL)	Date	Yield
7	Genetics	Should genetic testing that includes intronic sequencing be performed on infants with CRMS/CFSPID?	(((((((“CRMS”) OR (“CFTR Related Metabolic Syndrome”)) OR (“CFSPID”)) OR (“CF, Screen Positive Inconclusive Diagnosis”)) OR (“cystic fibrosis transmembrane conductance regulator/ genetics*”[All Fields]))) AND ((((“infant”[All Fields]) OR (“newborn”[All Fields])) OR (“neonatal screening”[All Fields])) OR (“newborn screening”[All Fields]))) AND ((genetic testing) OR (intronic sequencing))	3/29/2022	240	(“cystic fibrosis transmembrane conductance regulator - related metabolic syndrome” or “cystic fibrosis screen positive inconclusive diagnosis” or “CRMS” or “CFSPID” or “CFTR - related metabolic syndrome” or “cystic fibrosis transmembrane conductance regulator/genetic*” or “CFTR genetic*”) AND (“genetic testing” or “intronic sequencing” or infant or newborn) exclude medline	3/19/22	6
8	Genetics	What is the training or experience recommended for the HCP who is providing genetic counseling for families of infants with CRMS/ CFSPID? Merge with the communication PICO.	(((((((“CRMS”) OR (“CFTR Related Metabolic Syndrome”)) OR (“CFSPID”)) OR (“CF, Screen Positive Inconclusive Diagnosis”)) OR (“cystic fibrosis transmembrane conductance regulator/ genetics*”[All Fields]))) AND ((((“infant”[All Fields]) OR (“newborn”[All Fields])) OR (“neonatal screening”[All Fields])) OR (“newborn screening”[All Fields]))) AND ((communication) AND ((health personnel) OR (genetic counseling)))	3/30/2022	1	(“cystic fibrosis transmembrane conductance regulator - related metabolic syndrome” or “cystic fibrosis screen positive inconclusive diagnosis” or “CRMS” or “CFSPID” or “CFTR - related metabolic syndrome” or “cystic fibrosis transmembrane conductance regulator/genetic*” or “CFTR genetic*”) AND (“genetic counseling” or “health personnel” or “communication” or training or “genetic counselor” or credentialing or credentials or experience) exclude medline	3/19/22	5
9	Genetics	Should relatives (parents, siblings, and/or others) of infants with CRMS/CFSPID have *CFTR* evaluation?	(((((((“CRMS”) OR (“CFTR Related Metabolic Syndrome”)) OR (“CFSPID”)) OR (“CF, Screen Positive Inconclusive Diagnosis”)) OR (“cystic fibrosis transmembrane conductance regulator/ genetics*”[All Fields]))) AND ((((“infant”[All Fields]) OR (“newborn”[All Fields])) OR (“neonatal screening”[All Fields])) OR (“newborn screening”[All Fields]))) AND (((family) OR (relative)) AND ((((genetic testing) OR (evaluation)) OR (genetic screening)) OR (sweat test)))	3/30/2022	92	(“cystic fibrosis transmembrane conductance regulator - related metabolic syndrome” or “cystic fibrosis screen positive inconclusive diagnosis” or “CRMS” or “CFSPID” or “CFTR - related metabolic syndrome” or “cystic fibrosis transmembrane conductance regulator/genetic*” or “CFTR genetic*”) AND (“genetic testing” or evaluation or “genetic screening” or “sweat test” or “family evaluation”) exclude medline	3/19/22	15
10	Treatment	CRMS and respiratory culture positive for PA do you treat with	(((((((“CRMS”) OR (“CFTR Related Metabolic Syndrome”)) OR (“CFSPID”)) OR (“CF, Screen	3/30/2022	101	(“cystic fibrosis transmembrane conductance regulator - related metabolic syndrome” or “cystic	3/19/22	0

SUPPLEMENTAL TABLE 5 Continued

PICO	Group	Question	Search (PubMed)	Date	Yield	Search (CINAHL)	Date	Yield
		inhaled antibiotics? Do you flip to CF (include in the writing)?	Positive Inconclusive Diagnosis”)) OR (“cystic fibrosis transmembrane conductance regulator/ genetics*”[All Fields]))) AND ((((“infant”[All Fields]) OR (“newborn”[All Fields])) OR (“neonatal screening”[All Fields])) OR (“newborn screening”[All Fields]))) AND (((respiratory culture) OR (*Pseudomonas*)) OR (antibiotics))			fibrosis screen positive inconclusive diagnosis” or “CRMS” or “CFSPID” or “CFTR - related metabolic syndrome” or “cystic fibrosis transmembrane conductance regulator/genetic*” or “CFTR genetic*”) AND (“respiratory culture” or *Pseudomonas* or antibiotics or “pseudomonas aeruginosa” or “inhaled tobramycin” or tobi or colistin or eradication) exclude medline		
11	Treatment	CRMS and unexplained persistent cough (as defined by clinician $\leq$2 wk) do you treat with oral antibiotics (include this in education “when to call” for families)? Does this change with age?	(((((((“CRMS”) OR (“CFTR Related Metabolic Syndrome”)) OR (“CFSPID”)) OR (“CF, Screen Positive Inconclusive Diagnosis”)) OR (“cystic fibrosis transmembrane conductance regulator/ genetics*”[All Fields]))) AND ((((“infant”[All Fields]) OR (“newborn”[All Fields])) OR (“neonatal screening”[All Fields])) OR (“newborn screening”[All Fields]))) AND (((((((fluoroquinolone) OR (cephalosporin)) OR (aminoglycoside)) OR (β-lactam)) OR (carbapenem)) OR (antibiotic)) OR (oral antibiotic))	3/30/2022	17	(“cystic fibrosis transmembrane conductance regulator - related metabolic syndrome” or “cystic fibrosis screen positive inconclusive diagnosis” or “CRMS” or “CFSPID” or “CFTR - related metabolic syndrome” or “cystic fibrosis transmembrane conductance regulator/genetic*” or “CFTR genetic*”) AND (antibiotic* or fluoroquinolone or cephalosporin or aminoglycoside or β-lactam or carbapenem or “oral antibiotic*” or ciprofloxacin or levofloxacin or tobramycin or cough or “chronic cough” or exacerbation) exclude medline	3/19/22	0
12	Treatment	Should individuals with CRMS and a downward trajectory be on the standard diet or have calorie supplementation? Should this be split into 2 questions: one CF diet yes/no the other downward trajectory?	(((((((“CRMS”) OR (“CFTR Related Metabolic Syndrome”)) OR (“CFSPID”)) OR (“CF, Screen Positive Inconclusive Diagnosis”)) OR (“cystic fibrosis transmembrane conductance regulator/ genetics*”[All Fields]))) AND ((((“infant”[All Fields]) OR (“newborn”[All Fields])) OR (“neonatal screening”[All Fields])) OR (“newborn screening”[All Fields]))) AND (((standard diet) OR (caloric supplementation)))	3/30/2022	2	(“cystic fibrosis transmembrane conductance regulator - related metabolic syndrome” or “cystic fibrosis screen positive inconclusive diagnosis” or “CRMS” or “CFSPID” or “CFTR - related metabolic syndrome” or “cystic fibrosis transmembrane conductance regulator/genetic*” or “CFTR genetic*”) AND (“standard diet” or diet or “caloric supplementation” or calories or “nutritional failure” or “failure to thrive” or “weight loss” or “weight z scores” or growth or “oral supplement”) exclude medline	3/19/22	2

SUPPLEMENTAL TABLE 5 Continued

PICO	Group	Question	Search (PubMed)	Date	Yield	Search (CINAHL)	Date	Yield
13	Treatment	Should individuals with CRMS be on the standard salt intake or have salt supplementation? Depends on sweat test? What amount according to sweat test value?	(((((((“CRMS”) OR (“CFTR Related Metabolic Syndrome”)) OR (“CFSPID”)) OR (“CF, Screen Positive Inconclusive Diagnosis”)) OR (“cystic fibrosis transmembrane conductance regulator/ genetics*”[All Fields]))) AND ((((“infant”[All Fields]) OR (“newborn”[All Fields])) OR (“neonatal screening”[All Fields])) OR (“newborn screening”[All Fields]))) AND (((standard salt intake) OR (salt supplementation)) OR (sweat test))	3/30/2022	123	(“cystic fibrosis transmembrane conductance regulator - related metabolic syndrome” or “cystic fibrosis screen positive inconclusive diagnosis” or “CRMS” or “CFSPID” or “CFTR - related metabolic syndrome” or “cystic fibrosis transmembrane conductance regulator/genetic*” or “CFTR genetic*”) AND (salt or “standard salt intake” or “salt intake” or “salt supplementation” or “sweat test” or “sweat chloride” or hyponatremia or hyponatremic or “hyponatremic dehydration” or sodium or “sodium supplementation” or hypernatremia) exclude medline	3/19/22	4
14	Treatment	Should individuals with CRMS be on the standard vitamin intake or have CF specific vitamin supplementation? Move to monitoring: do we need to test (if we test then consider supplement)?	(((((((“CRMS”) OR (“CFTR Related Metabolic Syndrome”)) OR (“CFSPID”)) OR (“CF, Screen Positive Inconclusive Diagnosis”)) OR (“cystic fibrosis transmembrane conductance regulator/ genetics*”[All Fields]))) AND ((((“infant”[All Fields]) OR (“newborn”[All Fields])) OR (“neonatal screening”[All Fields])) OR (“newborn screening”[All Fields]))) AND ((((((((standard vitamin intake) OR (vitamin supplementation)) OR (vitamins)) OR (vitamin d)) OR (vitamin e)) OR (vitamin a)) OR (vitamin k)) OR (CF specific vitamin))	3/30/2022	13	(“cystic fibrosis transmembrane conductance regulator - related metabolic syndrome” or “cystic fibrosis screen positive inconclusive diagnosis” or “CRMS” or “CFSPID” or “CFTR - related metabolic syndrome” or “cystic fibrosis transmembrane conductance regulator/genetic*” or “CFTR genetic*”) AND (“standard vitamin intake” or “vitamin supplementation” or vitamin* or “vitamin d” or “vitamin e” or “vitamin a” or “vitamin k” or “CF specific vitamin” or “fat soluble vitamins” or “serum vitamin d” or “serum retinol” or “alpha-techoferol” or “vitamin d deficiency” or dexa or “bone disease”) exclude medline	3/19/22	0
15	Treatment	When should individuals with CRMS/ CFSPID who are eligible for modulators be on modulators? Other thoughts: should diagnosis be changed to CF OR do individuals with CRMS benefit from modulator treatment?	(((((((“CRMS”) OR (“CFTR Related Metabolic Syndrome”)) OR (“CFSPID”)) OR (“CF, Screen Positive Inconclusive Diagnosis”)) OR (“cystic fibrosis transmembrane conductance regulator/ genetics*”[All Fields]))) AND ((((“infant”[All Fields]) OR (“newborn”[All Fields])) OR (“neonatal screening”[All Fields])) OR (“newborn screening”[All	3/30/2022	20	(“cystic fibrosis transmembrane conductance regulator - related metabolic syndrome” or “cystic fibrosis screen positive inconclusive diagnosis” or “CRMS” or “CFSPID” or “CFTR - related metabolic syndrome” or “cystic fibrosis transmembrane conductance regulator/genetic*” or “CFTR genetic*”) AND (ivacaftor or lumacaftor or tezacaftor or	3/19/22	0

SUPPLEMENTAL TABLE 5 Continued

PICO	Group	Question	Search (PubMed)	Date	Yield	Search (CINAHL)	Date	Yield
			Fields])))) AND (((ivacaftor) OR (lumacaftor)) OR (tezacaftor))			elexacaftor or modulators) exclude medline		
16	Treatment	Should individuals with CRMS/CFSPID follow the CFF IPC guidelines? (ie, no contact with others with CF/ CRMS, school, clinic/hospital)	(((((((“CRMS”) OR (“CFTR Related Metabolic Syndrome”)) OR (“CFSPID”)) OR (“CF, Screen Positive Inconclusive Diagnosis”)) OR (“cystic fibrosis transmembrane conductance regulator/ genetics*”[All Fields]))) AND ((((“infant”[All Fields]) OR (“newborn”[All Fields])) OR (“neonatal screening”[All Fields])) OR (“newborn screening”[All Fields]))) AND (((ivacaftor) OR (lumacaftor)) OR (tezacaftor))	3/30/2022	20	(“cystic fibrosis transmembrane conductance regulator - related metabolic syndrome” or “cystic fibrosis screen positive inconclusive diagnosis” or “CRMS” or “CFSPID” or “CFTR - related metabolic syndrome” or “cystic fibrosis transmembrane conductance regulator/genetic*” or “CFTR genetic*”) AND (“infection prevention and control” or “infection control” or “infection protection” or cohorting or “bacterial transmission” or isolation or infection) exclude medline	3/19/22	0
17	Treatment	Should individuals with CRMS undergo ACT? Assess routine versus when sick.	(airway clearance therapy) AND (((((((“CRMS”) OR (“CFTR Related Metabolic Syndrome”)) OR (“CFSPID”)) OR (“CF, Screen Positive Inconclusive Diagnosis”)) OR (“cystic fibrosis transmembrane conductance regulator/ genetics*”[All Fields]))) AND ((((“infant”[All Fields]) OR (“newborn”[All Fields])) OR (“neonatal screening”[All Fields])) OR (“newborn screening”[All Fields])))	3/30/2022	5	(“cystic fibrosis transmembrane conductance regulator - related metabolic syndrome” or “cystic fibrosis screen positive inconclusive diagnosis” or “CRMS” or “CFSPID” or “CFTR - related metabolic syndrome” or “cystic fibrosis transmembrane conductance regulator/genetic*” or “CFTR genetic*”) AND (“airway clearance” or “airway clearance techniques” or “airway clearance therapy” or “sputum clearance techniques” or “act” or “chest physiotherapy” or “high frequency chest wall oscillation device” or VEST or “chest percussion” or “postural drainage” or exercise or “respiratory therapy”) exclude medline	3/19/22	3
18	Treatment	Should individuals with CRMS/CFSPID use CF specific medications (hypertonic, dornase alfa)?	(((((((“CRMS”) OR (“CFTR Related Metabolic Syndrome”)) OR (“CFSPID”)) OR (“CF, Screen Positive Inconclusive Diagnosis”)) OR (“cystic fibrosis transmembrane conductance regulator/ genetics*”[All Fields]))) AND ((((“infant”[All Fields]) OR (“newborn”[All Fields])) OR (“neonatal screening”[All Fields]))	3/30/2022	3	(“cystic fibrosis transmembrane conductance regulator - related metabolic syndrome” or “cystic fibrosis screen positive inconclusive diagnosis” or “CRMS” or “CFSPID” or “CFTR - related metabolic syndrome” or “cystic fibrosis transmembrane conductance regulator/genetic*” or “CFTR genetic*”) AND (“hypertonic saline” or “dornase alfa” or	3/19/22	0

**SUPPLEMENTAL TABLE 5** Continued								
PICO	Group	Question	Search (PubMed)	Date	Yield	Search (CINAHL)	Date	Yield
			OR (“newborn screening”[All Fields]))) AND ((hypertonic saline) OR (dornase alfa))			pulmozyme or enzymes or “lipase enzymes” or pancrealipase or “pancreatic enzyme replacement therapy” or PERT or albuterol or mucomyst or acetylcysteine) exclude medline		
19	Monitoring	Which team members should see CRMS individuals? Make consistent with other PICO (ie, MHC for questions asked). Include with the education question?	((((((((“CRMS”) OR (“CFTR Related Metabolic Syndrome”)) OR (“CFSPID”)) OR (“CF, Screen Positive Inconclusive Diagnosis”)) OR (“cystic fibrosis transmembrane conductance regulator/ genetics*”[All Fields]))) AND ((((“infant”[All Fields]) OR (“newborn”[All Fields])) OR (“neonatal screening”[All Fields])) OR (“newborn screening”[All Fields]))) AND (((((((((nutritionist) OR (dietitian)) OR (respiratory therapist)) OR (psychologist)) OR (psychiatrist)) OR (social worker)) OR (physical therapist)) OR (pharmacist)) OR (nurse))	3/30/2022	16	(“cystic fibrosis transmembrane conductance regulator - related metabolic syndrome” or “cystic fibrosis screen positive inconclusive diagnosis” or “CRMS” or “CFSPID” or “CFTR - related metabolic syndrome” or “cystic fibrosis transmembrane conductance regulator/genetic*” or “CFTR genetic*”) AND (nutritionist or dietician or nutrition or “respiratory therapist” or psychologist or psychiatrist or “social worker” or “physical therapist” or pharmacist or nurse or “mental health” or “genetic counseling” or pulmonologist) exclude medline	3/19/22	1
20	Monitoring	Should routine respiratory cultures be obtained in individuals with CRMS, and how often?	(respiratory culture) AND (((((((“CRMS”) OR (“CFTR Related Metabolic Syndrome”)) OR (“CFSPID”)) OR (“CF, Screen Positive Inconclusive Diagnosis”)) OR (“cystic fibrosis transmembrane conductance regulator/ genetics*”[All Fields]))) AND ((((“infant”[All Fields]) OR (“newborn”[All Fields])) OR (“neonatal screening”[All Fields])) OR (“newborn screening”[All Fields])))	3/30/2022	36	(“cystic fibrosis transmembrane conductance regulator - related metabolic syndrome” or “cystic fibrosis screen positive inconclusive diagnosis” or “CRMS” or “CFSPID” or “CFTR - related metabolic syndrome” or “cystic fibrosis transmembrane conductance regulator/genetic*” or “CFTR genetic*”) AND (“respiratory culture” or culture or “respiratory therapy” or “expectorated sputum” or “bacterial culture” or “respiratory microbiology” or “broncheoalveolar lavage” or “double gag culture” or “throat culture” or “induce sputum” or “pseudomonas aeruginosa”) exclude medline	3/19/22	0
21	Monitoring	How often should individuals with CRMS have routine imaging testing done?	(((((((“CRMS”) OR (“CFTR Related Metabolic Syndrome”)) OR (“CFSPID”)) OR (“CF, Screen Positive Inconclusive Diagnosis”)) OR (“cystic fibrosis	3/30/2022	49	(“cystic fibrosis transmembrane conductance regulator - related metabolic syndrome” or “cystic fibrosis screen positive inconclusive	3/19/22	0

SUPPLEMENTAL TABLE 5 Continued

PICO	Group	Question	Search (PubMed)	Date	Yield	Search (CINAHL)	Date	Yield
			transmembrane conductance regulator/ genetics*"[All Fields]))) AND ((((“infant”[All Fields]) OR (“newborn”[All Fields])) OR (“neonatal screening”[All Fields])) OR (“newborn screening”[All Fields]))) AND (((((((((imaging) OR (imaging testing)) OR (x-ray)) OR (CT scan)) OR (MRI)) OR (Ultrasound)) OR (x-ray computed tomography)) OR (ultrasonography)) OR (MRI))			diagnosis” or “CRMS” or “CFSPID” or “CFTR - related metabolic syndrome” or “cystic fibrosis transmembrane conductance regulator/genetic*” or “CFTR genetic*”) AND (imaging or radiography or radiology or “imaging testing” or x-ray or CT scan or MRI or ultrasound or “x-ray computed tomography” or ultrasonography or “magnetic resonance imaging”) exclude medline		
22	Monitoring	How often should sweat testing be done; does initial level or increase in sweat [Cl^-] play a role? If and when should we stop (age)? If normal sweat test, should it be repeated? Is it mutation specific? If normal, what age do you stop if you keep screening?	(((((((“CRMS”) OR (“CFTR Related Metabolic Syndrome”)) OR (“CFSPID”)) OR (“CF, Screen Positive Inconclusive Diagnosis”)) OR (“cystic fibrosis transmembrane conductance regulator/ genetics*”[All Fields]))) AND ((((“infant”[All Fields]) OR (“newborn”[All Fields])) OR (“neonatal screening”[All Fields])) OR (“newborn screening”[All Fields]))) AND ((((sweat) OR (sweat testing)) OR (chloride)) OR (chloride concentration))	3/30/2022	286	(“cystic fibrosis transmembrane conductance regulator - related metabolic syndrome” or “cystic fibrosis screen positive inconclusive diagnosis” or “CRMS” or “CFSPID” or “CFTR - related metabolic syndrome” or “cystic fibrosis transmembrane conductance regulator/genetic*” or “CFTR genetic*”) AND (sweat or “sweat test” or “sweat chloride test” or “sweat testing” or “chloride concentration” or chloride or “pilocarpine iontophoresis” or “sweat chloride” or nanoduct or macroduct) exclude medline	3/19/22	3
23	Monitoring	Should pulmonary function laboratory studies be done and which ones? (ensure consistency with treatment PICOs; PFT, LCI etc.) Should that be its own question? Functional testing of CFTR? If laboratory tests are normal, do you need to keep following?	(((((((“CRMS”) OR (“CFTR Related Metabolic Syndrome”)) OR (“CFSPID”)) OR (“CF, Screen Positive Inconclusive Diagnosis”)) OR (“cystic fibrosis transmembrane conductance regulator/ genetics*”[All Fields]))) AND ((((“infant”[All Fields]) OR (“newborn”[All Fields])) OR (“neonatal screening”[All Fields])) OR (“newborn screening”[All Fields]))) AND (((((pulmonary testing) OR (pulmonary function testing)) OR (PFT)) OR (LCI)) OR (lung clearance index))	3/30/2022	101	(“cystic fibrosis transmembrane conductance regulator - related metabolic syndrome” or “cystic fibrosis screen positive inconclusive diagnosis” or “CRMS” or “CFSPID” or “CFTR - related metabolic syndrome” or “cystic fibrosis transmembrane conductance regulator/genetic*” or “CFTR genetic*”) AND (“pulmonary testing” or “pulmonary function testing” or PFT or LCI or MBW or “lung clearance index” or “multiple breath washout” or “lung function testing” or spirometry or FEV1 or	3/19/22	1

SUPPLEMENTAL TABLE 5 Continued

PICO	Group	Question	Search (PubMed)	Date	Yield	Search (CINAHL)	Date	Yield
						“forced expiratory volume in 1 second” or “infant PFT” or “lung volume” or plethysmography or “nitrogen washout”) exclude medline		
24	Monitoring	Should chemistry laboratory studies be done and which ones (ensure consistency with treatment PICOs)? Functional testing of CFTR? If laboratory tests are normal, do you need to keep following?	(((((((“CRMS”) OR (“CFTR Related Metabolic Syndrome”)) OR (“CFSPID”)) OR (“CF, Screen Positive Inconclusive Diagnosis”)) OR (“cystic fibrosis transmembrane conductance regulator/ genetics*”[All Fields]))) AND ((((“infant”[All Fields]) OR (“newborn”[All Fields])) OR (“neonatal screening”[All Fields])) OR (“newborn screening”[All Fields]))) AND (((((((((((((((((bilirubin) OR (ast)) OR (alt)) OR (ggt)) OR (platelet count)) OR (comprehensive metabolic)) OR (vitamin a)) OR (vitamin d)) OR (vitamin e)) OR (vitamin k)) OR (fecal elastase)) OR (pancreatic elastase)) OR (stool elastase)) OR (CBC)) OR (IgE))) OR (Hemoglobin A1C)) OR (oral glucose tolerance test))	3/31/2022	38	(“cystic fibrosis transmembrane conductance regulator - related metabolic syndrome” or “cystic fibrosis screen positive inconclusive diagnosis” or “CRMS” or “CFSPID” or “CFTR - related metabolic syndrome” or “cystic fibrosis transmembrane conductance regulator/genetic*” or “CFTR genetic*”) AND (bloodwork or “liver function test” or bilirubin or ast or alt or ggt or platelet or CMP or “comprehensive metabolic” or “vitamin a” or “vitamin e” or “vitamin d” or “vitamin k” or “fecal elastase” or “pancreatic elastase” or “stool elastase” or cbc or “complete blood count” or ige or “hemoglobin A1C” or “oral glucose tolerance test”) exclude medline	3/19/22	0

CINHAL, Cumulated index to nursing and allied health literature; PA, Pseudomonas aeruginosa.
Supplemental References

SUPPLEMENTAL TABLE 6 Genetic Glossary of Terms

Term		Definition
Exon		A segment of DNA that codes for a protein; exons are often referred to as the "coding region" and are always included in a clinical *CFTR* sequencing test.
Flanking region		A limited number of intronic DNA base pairs (often 10–50) that lie immediately adjacent to the exons; these small portions of the intron are typically included in a clinical *CFTR* sequencing test performed.
In *cis*		Two or more *CFTR* variants existing in the same copy of the *CFTR* gene; variants in *cis* have been inherited from the same parent.
In *trans*		Two variants existing in different (opposite) *CFTR* genes; variants in *trans* have been inherited from both parents.
Intron		A segment of DNA that does not code for a protein; introns are often referred to as the "noncoding region" and may not be included in a clinical *CFTR* sequencing test unless specified.
CFTR genetic testing		A method of evaluating the *CFTR* gene for DNA changes (variants).
	CFTR panel testing (also called DNA panel or variant panel)	A genetic test that determines the presence or absence of specific *CFTR* variants. Panel testing will evaluate only for variants on a predetermined list, typically composed of common CF-causing variants. Panels may vary in size from 23 to >100 variants and their sensitivity varies by the ancestry of the person being tested. A DNA variant not included on the panel will not be evaluated or reported if present.
	CFTR sequencing	A genetic test that determines the order of nucleotides in *CFTR* DNA. Sequencing may be performed using a variety of methods and may include the entire *CFTR* gene or limited portions only, such as of the coding region.
	Sanger sequencing	A specific method used to perform DNA sequencing. This method is usually used on a single gene (such as *CFTR*) to find CF-causing variants in a person being evaluated for CF. This method typically evaluates the exons of *CFTR* and a small portion of the introns (the flanking region). Large deletions and duplications, such as the variant CFTRdele2,3, may not be found by sequencing tests. Sanger sequencing is a more traditional way of performing DNA sequencing and may have somewhat higher cost and longer result time.
	Next-generation sequencing (next-gen or NGS)	A newer technology to determine the sequence of nucleotides in DNA. This method may be used on a single gene (such as *CFTR*) or on many genes (such as exome sequencing or genome sequencing). NGS became more widespread in the mid-2010s and is now the main technology used in diagnostic *CFTR* testing. Large deletions and duplications, such as the variant CFTRdele2,3, may be found by NGS. However, finding these types of variants may require a different type of analysis. NGS may have a somewhat lower cost and a shorter result time than Sanger sequencing.
	Full-gene *CFTR* sequencing	A sequencing test that looks at the entire *CFTR* gene and includes all exons and all portions of all introns. This is a new test that is different from the typical *CFTR* sequencing test that has been used over the past decades. This test may also be called whole-gene *CFTR* sequencing and typically uses next-generation sequencing technology.
	Intronic sequencing	A sequencing test that includes the noncoding (intronic) regions of *CFTR*. Typically, intronic sequencing is not ordered as a separate test, but is included when ordering full-gene *CFTR* sequencing. It is important to distinguish whether a test has included sequencing of the flanking region only (which is only a small portion of the introns and often included when sequencing just the coding region of the gene) or has evaluated the full intronic sequence.
	Del/dup testing	An evaluation for large structural variants that typically include 1 or more exons (eg, CFTRdele2,3, which is a deletion of exons 2 and 3 in *CFTR*). Large deletions and duplications involving exons cannot be detected by Sanger sequencing and may require an additional test, such as multiplex ligation-dependent probe amplification. Some NGS tests can detect large deletions and duplications involving exons, but additional analysis might be needed. It is important to note whether a *CFTR* sequencing test will include del/dup analysis or whether this requires a separate test order.
Variant interpretation		A prediction of the molecular and/or phenotypic consequence of a given DNA variant. Variant interpretations according to CFTR2 (https://cftr2.org) are provided below.
	CF-causing variant	A *CFTR* variant that is expected to cause CF when found in *trans* with another CF-causing variant.
	Non-CF-causing variant	A *CFTR* variant that is NOT expected to cause CF, even when found in *trans* with a CF-causing variant. Some individuals with CF have non-CF-causing variants, but it is expected that these variants are not the cause of disease. Non-CF-causing variants may be present in addition to 2 CF-causing variants.
	VVCCs	A *CFTR* variant that may result in CF in some people but not in others, when found in *trans* with a CF-causing variant. Individuals with VVCCs who do not have CF may have a CFTR-related disorder or no symptoms.
	Variant of unknown significance	A *CFTR* variant for which the clinical significance is not clear. Over time and as more data are collected, some VUS -may be reinterpreted as CF-causing, non-CF-causing, or VVCCs. Clinicians are recommended to review VUS regularly to see if the interpretation has changed
Exome sequencing		A testing method that looks at almost every nucleotide in the coding region (exons) of all genes in the body, including *CFTR*
Genome sequencing		A testing method that looks at almost every nucleotide in a person's entire DNA sequence. This test includes both the coding (exons) and noncoding (introns) regions of all genes, including *CFTR*.

NGS, next-generation sequencing, VUS, variant of unknown significance.

SUPPLEMENTAL REFERENCES

84. Quigley SJ, Linnane B, Connellan S, et al. Psychosocial distress and knowledge deficiencies in parents of children in Ireland who carry an altered cystic fibrosis gene. *J Genet Couns*. 2018;27(3):589–596

85. La Pean A, Collins JL, Christopher SA, et al. A qualitative secondary evaluation of statewide follow-up interviews for abnormal newborn screening results for cystic fibrosis and sickle cell hemoglobinopathy. *Genet Med*. 2012;14(2):207–214

86. Barben J, Chudleigh J. Processing newborn bloodspot screening results for CF. *Int J Neonatal Screen*. 2020;6(2):25
